# Gut microbiota profiles and potential biomarkers in pre-pubertal females with ADHD

**DOI:** 10.3389/fmicb.2026.1894383

**Published:** 2026-08-20

**Authors:** Guanjun Bao, Suxian Tan, Yiming An, Ye Luo

**Affiliations:** 1 Quzhou College of Technical, School of Medicine, Quzhou, China; 2 Quzhou City Third Hospital, Quzhou, China; 3 Faculty of Basic Medicine, Department of Physiology, Daqing Medical College, Daqing, China

**Keywords:** attention deficit hyperactivity disorder, females, gut microbiota, microbiota-gut-brain axis, pre-pubertal

## Abstract

**Background:**

Attention-deficit/hyperactivity disorder (ADHD) is a highly prevalent neurodevelopmental disorder with significant sex differences in diagnostic rates; males being diagnosed more frequently than females. Gut microbiota alterations may contribute to ADHD via the microbiota-gut-brain axis. However, previous microbiota studies have predominantly used male or mixed-gender samples, neglecting females with distinct clinical and hormonal profiles. The pre-pubertal period is critical for both neurodevelopment and microbiota maturation. This study investigates gut microbiota characteristics in pre-pubertal females with ADHD to identify sex-specific biomarkers and candidate targets for future investigation.

**Aim of the study:**

This study aimed to characterize gut microbiota profiles in pre-pubertal females with ADHD using 16S rRNA sequencing, and to identify differentially abundant taxa and potential biomarkers through LEfSe and MetagenomeSeq analyses.

**Methods and materials:**

Pre-pubertal female ADHD patients (DSM-5 diagnosed, drug-naïve) were recruited from Quzhou Third Hospital. Fecal DNA was extracted, the 16S rRNA gene amplified by PCR, and libraries sequenced on the Revio platform. Bioinformatics analysis included diversity assessment and differential abundance analysis using MetagenomeSeq and LEfSe methods.

**Results:**

Alpha-diversity analysis revealed reduced community evenness (lower Shannon and Simpson indices) but preserved species richness (comparable Chao1) in the ADHD group. Beta-diversity analysis showed significant group differences for both Weighted and Unweighted UniFrac (PERMANOVA, *P* < 0.01), with a larger effect size for Unweighted UniFrac, suggesting that rare taxa may substantially contribute to community alterations. MetagenomeSeq identified *Verrucomicrobiota* as the only significantly elevated phylum in ADHD. LEfSe distinguished 26 differential taxa, with cross-validation designating *Ruminococcus* (enriched in ADHD) and *Coprobacillus* (depleted in ADHD) as candidate biomarkers.

**Conclusion:**

Pre-pubertal females with ADHD exhibited reduced gut bacterial evenness with preserved richness, characterized by higher *Ruminococcus* and lower *Coprobacillus*, suggesting lineage-specific reshuffling rather than wholesale species loss. These findings implicate gut microbiota community alterations in ADHD pathogenesis via the microbiota-gut-brain axis.

## 1 Introduction

Attention deficit hyperactivity disorder (ADHD) is a highly prevalent neurodevelopmental disorder characterized by inattention, hyperactivity, and impulsivity that are inconsistent with the individual’s developmental level (Faraone et al., 2024; Durdurak et al., 2025). Epidemiological data indicate that the global pooled prevalence of ADHD is approximately 7.2%, with a significantly higher diagnostic rate in males than in females (Huang and Liu, 2025; Osianlis et al., 2026). This sex difference may be partly attributable to variations in clinical presentation between sexes: males more frequently exhibit typical externalizing symptoms such as hyperactivity and impulsivity, whereas females tend to present with internalizing features, predominantly inattention (Babinski, 2024; Osianlis et al., 2026). Notably, the early conceptualization of ADHD and the majority of available assessment tools were established based on externalizing behaviors centered around hyperactivity, which may introduce diagnostic bias against female patients and consequently obscure genuine sex differences in the clinical manifestation of ADHD (Bruchmüller et al., 2012; Cortese et al., 2023; Osianlis et al., 2026). Furthermore, although the prevalence of ADHD is generally higher in males during childhood and adolescence, the prevalence in females tends to exceed that in males in the adult population – a phenomenon that may be associated with fluctuations in female hormone levels across different life stages (Bruchmüller et al., 2012; Babinski, 2024; Rosenthal and Hinshaw, 2024; Durdurak et al., 2025; Osianlis et al., 2026).

As one of the most common neurodevelopmental disorders with childhood onset, ADHD is characterized by early emergence, chronic persistence, and extensive functional impairment across academic, occupational, social, and behavioral domains, including increased risk of accidents and substance use disorders (de Schipper et al., 2015; Holst and Thorell, 2020; Golmirzaei et al., 2016). Longitudinal research indicates that its developmental trajectories can be classified into at least four subtypes: early-onset preschool type (ages 3–5 years), childhood-onset persistent type (ages 6–14 years), childhood-onset remitting type with adolescent resolution childhood-onset remitting type with symptom resolution during adolescence (defined as onset before age 12 with remission to subthreshold or full remission levels sustained through adolescent follow-up, typically assessed between ages 12–18 years), and adolescent/adult-onset type (≥16 years) (Posner et al., 2020; Van Meter et al., 2024; Liu et al., 2025). This heterogeneity is manifested not only in disease course but also across multiple domains of functional impairment; ADHD rarely affects a single functional area in isolation. Instead, it frequently co-occurs with various psychiatric comorbidities, exerting sustained and profound adverse effects on patients’ physical health, academic performance, interpersonal relationships, and occupational development (Gilsbach et al., 2025; Ryszkiewicz et al., 2025; Silva Santos et al., 2025). Given its chronic trajectory and multidimensional functional deficits, ADHD and its intervention strategies have garnered increasing attention from both clinical and public health perspectives.

The pathophysiological mechanisms underlying ADHD are not governed by a single dominant factor but rather arise from the interplay among genetic susceptibility, environmental exposures, and neurobiological processes (Anand et al., 2017). Although studies have demonstrated heritability estimates of 70%–90% for ADHD, accumulating evidence indicates that environmental factors also play a non-negligible role in its etiology and progression (Faraone and Larsson, 2019). Notably, genetically susceptible individuals may exhibit heightened sensitivity to specific environmental risks, with both factors influencing disease phenotypes through complex gene-environment interactions (Wang et al., 2020; Panpetch et al., 2024). Among various environmental influences, dietary patterns during pregnancy and early life have garnered increasing scientific attention. Research suggests that these factors may induce microbial community alterations by altering intestinal permeability and modulating microbiota composition and metabolite production (Cassidy-Bushrow et al., 2023; Wang et al., 2025). These findings implicate gastrointestinal microecological imbalance as a potentially critical intermediary linking early environmental exposures to ADHD pathogenesis risk.

Recent advances in microbiota-gut-brain axis research have provided mechanistic support for these associations (Góralczyk-Bińkowska et al., 2022; Wang et al., 2023; Gilsbach et al., 2025). Substantial evidence indicates that intestinal microbiota can influence brain structure and function through immunological, endocrine, and neural pathways (Yassin et al., 2025). Germ-free animal model studies have demonstrated that the absence of intestinal microbiota leads to aberrant brain development, whereas interventions with specific bacterial strains can induce behavioral modifications and exert protective effects against certain neurological disorders (Parracho et al., 2005; Cheng et al., 2020; Richarte et al., 2021; Dargenio et al., 2023). Furthermore, multiple experiments have shown that alterations in gut microbiota composition are associated with changes in anxiety-like and social behaviors, often accompanied by fluctuations in neurotransmitter levels within emotion-related brain regions such as the prefrontal cortex and amygdala (Shoji et al., 2023; Deng et al., 2021; Needham et al., 2022). These observations suggest that microbiota may shape host behavioral phenotypes by regulating local and central neurotransmitter metabolism. Although direct evidence in human studies remains limited, preliminary data indicate associations between gut microbiota compositional characteristics and individual emotion regulation capacities, potentially mediated by their influence on dopamine and serotonin release (Roman et al., 2018; Painold et al., 2019; Houtman et al., 2022). Given that dysregulation of dopaminergic and serotonergic systems is one of the core pathophysiological mechanisms implicated in ADHD, exploring the relationship between gut microbiota composition and these neurotransmitter pathways may provide novel insights into the neurobiological basis of the disorder. However, the specific gut microbial taxa associated with ADHD and their functional links to neurotransmitter metabolism remain largely unexplored (Wan et al., 2020; Predescu et al., 2024; Dias et al., 2025; Solomon et al., 2025). Collectively, these studies converge on a critical hypothesis: alterations in intestinal microbiota may play a significant role in ADHD pathogenesis through their effects on central nervous system function.

Notably, female ADHD patients exhibit unique characteristics regarding clinical symptomatology, diagnostic bias, and hormonal influences, yet previous gut microbiota studies have predominantly employed male or mixed-gender samples, with research specifically targeting female populations being extremely scarce (Babinski, 2024; Osianlis et al., 2026; Wynchank et al., 2026). Moreover, the pre-pubertal period represents a critical window for both neurodevelopment and gut microbiota maturation; microbiota characteristics during this stage may provide unique insights into early disease mechanisms (Romeo, 2013; McVey Neufeld et al., 2016; Esposito and Ismail, 2022; Carson et al., 2023; Nankova et al., 2025). Therefore, investigating gut microbiota in pre-pubertal female ADHD patients – a specific subgroup – may offer novel entry points for elucidating disease heterogeneity, identifying early biomarkers, and developing microbiota-targeted intervention strategies.

Based on these considerations, the present study aims to focus on pre-pubertal female ADHD patients and explore ADHD-associated microbiota profiles by comparing their gut microbiota compositional characteristics with those of healthy controls. This investigation seeks to provide scientific evidence for clarifying sex-specific pathogenic mechanisms of ADHD, identifying potential early predictive biomarkers, and developing microbiota-targeted adjunctive interventions.

## 2 Subjects and methods

This study protocol has been approved by the Institutional Review Board (IRB) of Quzhou Third Hospital and the Ethics Committee of the Medical School at Quzhou College of Technical. The research process strictly adheres to the guiding principles of the Declaration of Tokyo, the Declaration of Helsinki, Good Clinical Practice (GCP) guidelines, and relevant laws and regulations. All eligible participants and their legal guardians have been fully informed of the study’s objectives, procedures, and potential risks, and have provided written informed consent.

### 2.1 Study design and participants

Participants in the ADHD group were recruited from the pediatric psychiatry and neurology wards of Quzhou Third Hospital. The inclusion criteria for ADHD patients were as follows: (1) a confirmed diagnosis of ADHD by a child and adolescent psychiatrist according to the Diagnostic and Statistical Manual of Mental Disorders, Fifth Edition (DSM-5), with intellectual functioning assessed by a licensed clinical psychologist using the Wechsler Intelligence Scale for Children; (2) female sex and pre-pubertal status (i.e., menarche not yet achieved); and (3) no prior exposure to any anti-ADHD pharmacotherapy.

The exclusion criteria included: (1) previous or current diagnosis of any neurological or psychiatric disorder; (2) chronic systemic diseases (e.g., diabetes, autoimmune disorders, cardiovascular disease, hepatic or renal dysfunction); (3) use of antibiotics or probiotics within the past 4 weeks; (4) acute infection or gastrointestinal illness within the past 4 weeks; (5) use of medications known to affect central nervous system or gastrointestinal function (e.g., antipsychotics, antidepressants); and (6) special diets (e.g., vegetarian, gluten-free diets).

**FIGURE 1:**
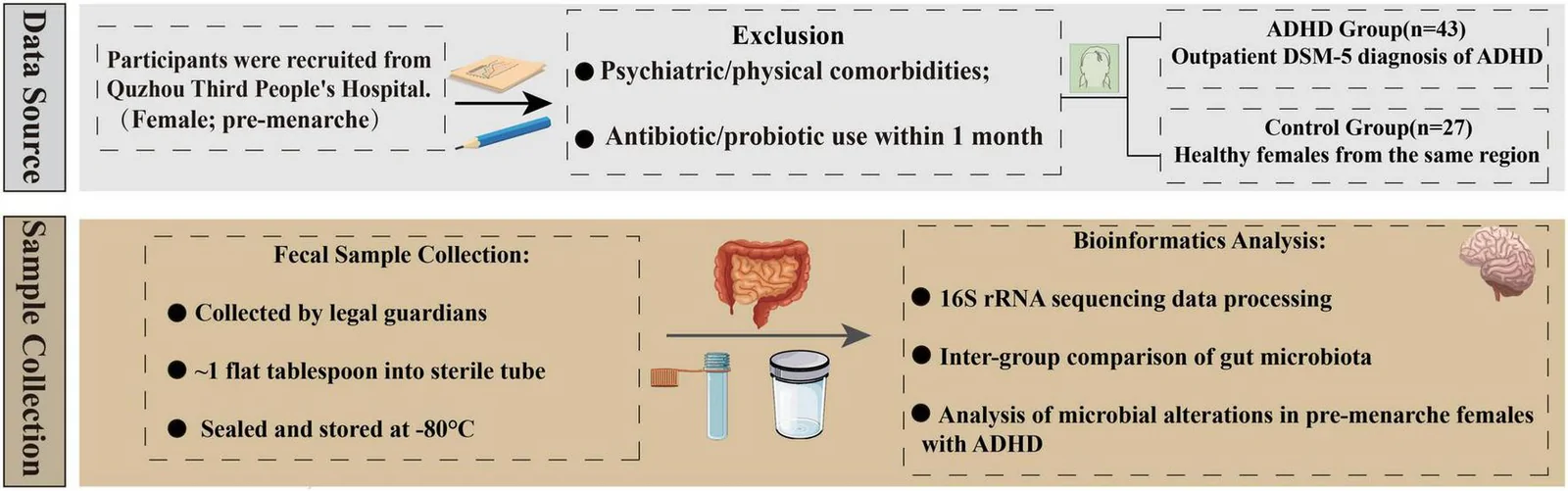
Experimental procedure flowchart.

Healthy controls were selected from pre-pubertal girls residing in the same urban district who had not yet reached menarche. ADHD participants and healthy controls were matched as closely as possible by age (±6 months) and sex, with each control matched to an ADHD participant of the same sex and closest age. This strategy aimed to minimize confounding by age and sex. Due to recruitment constraints, however, the final sample sizes were unequal, with 43 ADHD participants and 27 healthy controls included in the final analytical cohort (Figure 1).

### 2.2 Sample collection

Fecal samples were collected with the assistance of the children’s legal guardians. Approximately 5 g of feces was transferred into a sterile plastic tube with a secure sealing cap. Samples were collected within 30 min of defecation and immediately placed at 4 °C. They were transported to the laboratory within 2 h in insulated boxes with ice packs, where they were aliquoted and frozen at −80 °C until DNA extraction. Storage duration prior to extraction ranged from 1 to 6 months (median ∼3 months). Samples were not subjected to freeze-thaw cycles.

### 2.3 16 S rRNA gene amplicon sequencing

Fecal DNA was extracted using the BeyoMag™ Stool Genomic DNA Purification Kit with Magnetic Beads (BeyoMag). The V4 region of the bacterial 16S rRNA gene was amplified using barcoded primers 515F (5′-CCTAYGGGRBGCASCAG-3′) and 806R (5′-GGACTACNNGGGTATCTAAT-3′) in 15 μL reactions containing Phusion High-Fidelity PCR Master Mix, 0.2 μM each primer, and ∼10 ng template DNA. Cycling conditions: 98 °C for 1 min; 30 cycles of 98 °C for 10 s, 50 °C for 30 s, and 72 °C for 30 s; final extension at 72 °C for 5 min. Amplicons were subjected to end-repair, A-tailing, adapter ligation, and double magnetic bead purification. Libraries were sequenced on the Illumina NovaSeq platform (PE250).

Raw paired-end reads were merged using FLASH (v1.2.11) and quality-filtered using fastp (v0.23.1). ASVs were inferred using the DADA2 plugin in QIIME2 (v2025.4) with concurrent chimera removal. The feature table was rarefied to 61,776 reads per sample. Taxonomic assignment was performed using the feature-classifier plugin against the SILVA 138.1 database.

### 2.4 Microbiome statistical analyses

To enhance the robustness of differential taxon identification and reduce method-dependent false positives, we employed a cross-validation strategy at the genus level, integrating multiple complementary statistical approaches. Specifically, the Wilcoxon rank-sum test served as the primary non-parametric method for two-group comparisons, given the non-normal distribution of most microbiome abundance data. Student’s *t*-test was additionally applied to normally distributed subset data as a complementary parametric validation. Furthermore, the zero-inflated Gaussian model implemented in metagenomeSeq – which is specifically designed for sparse, zero-inflated microbiome count data – was used for phylum-level differential abundance screening and, at the genus level, served as a third independent validation tool to cross-validate the findings from the Wilcoxon and *t*-test analyses.

Given the non-normal distribution of microbiome data, non-parametric methods were applied. Alpha-diversity indices were compared using the Wilcoxon rank-sum test. Beta diversity was evaluated by PERMANOVA (999 permutations) on weighted and unweighted UniFrac distances, with betadisper test for dispersion homogeneity. Differential abundance at the phylum level was analyzed using metagenomeSeq (zero-inflated Gaussian model), with significance defined as |log_2_FC| > 1 and FDR-adjusted *q* < 0.05 (Benjamini–Hochberg). LEfSe (LDA > 2, *P* < 0.05) was used for multi-level differential taxon discovery. Key genera were validated using Wilcoxon rank-sum tests with FDR correction. All tests were two-sided; *P* < 0.05 or *q* < 0.05 was considered significant.

## 3 Results

### 3.1 Statistical analysis

A total of 70 participants who met the inclusion criteria were recruited, comprising 43 girls with ADHD (pre-pubertal) and 27 typically developing (TD) healthy children matched for age and sex, all residing in the same urban district. The characteristics distinguishing the ADHD group from the healthy control group are summarized in Table 1.

**TABLE 1:** Comparison between pre-pubertal Girls with ADHD and typically developing healthy controls

Characteristics	ADHD (*n* = 43)	Healthy children (*n* = 27)	*P*-value
Age (year)	9.74 ± 2.39	9.48 ± 2.59	0.675
BMI (kg/m^2^)	17.33 ± 3.95	17.30 ± 3.82	0.975
Total ADHD symptom score	23.02 ± 2.98	6.19 ± 1.93	<0.001

### 3.2 Microbial diversity analysis

**FIGURE 2:**
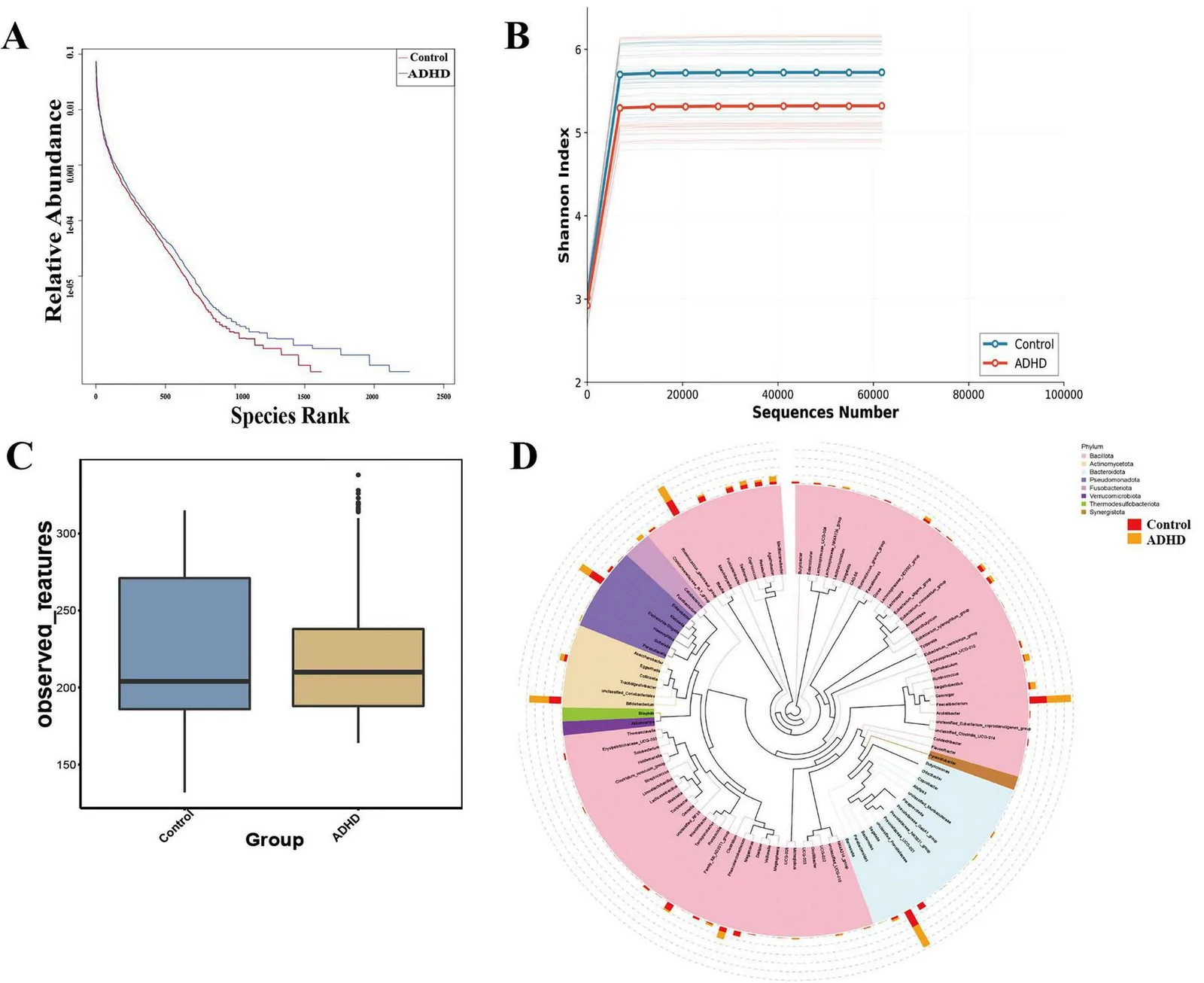
Comparison of gut microbiome characteristics between ADHD patients and healthy controls. **(A)** Species rank curve showing the species abundance distribution across samples in both groups; **(B)** Rarefaction curve reflecting the relationship between sequencing depth and species richness; **(C)** Boxplot of observed species comparing alpha diversity between the two groups; **(D)** Linear discriminant analysis effect size (LEfSe) cladogram displaying differentially abundant taxa between groups (LDA > 2, *P* < 0.05), with red indicating control-enriched taxa and yellow indicating ADHD-enriched taxa.

We performed fecal microbiome sequencing on all subjects. A total of 15,703,871 raw paired-end reads (Raw PE) were generated, with an average of 104,692 reads per sample (range: 67,535–108,055). After quality filtering and paired-end merging, 15,596,326 high-quality tags were retained, averaging 103,976 tags per sample, representing a retention rate of 99.31%. To eliminate the effect of uneven sequencing depth on downstream diversity comparisons, all samples were randomly subsampled to a uniform depth of 61,776 reads prior to analysis. The rank-abundance curves exhibited a typical long-tail distribution in both groups, indicating that a small number of dominant species accounted for the vast m1ajority of abundance while the vast majority of species were extremely rare; the ADHD group curve extended further into the low-abundance region with slight elevation at intermediate ranks, suggesting an increased number of rare species at negligible abundances and a polarized distribution with reduced community evenness (Figure 2A). Rarefaction analysis was performed to assess whether the sequencing depth was sufficient to capture the microbial diversity in each sample. The Shannon diversity index rarefaction curves for all 70 samples (43 controls and 27 ADHD cases) plateaued rapidly and reached saturation at a sequencing depth of approximately 7,000 reads (Figure 2B).

**FIGURE 3:**
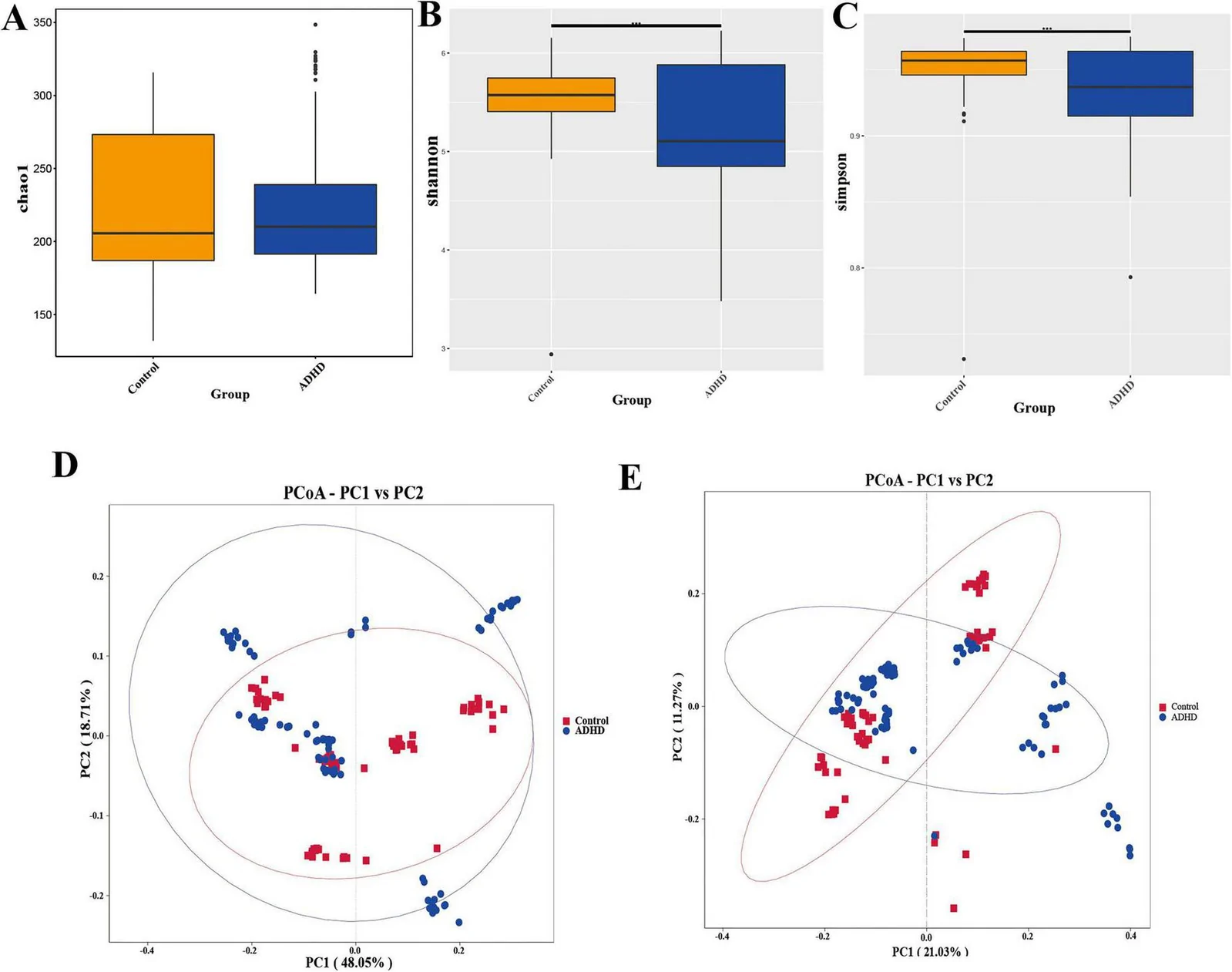
Comparison of alpha diversity and beta diversity of gut microbiome between ADHD patients and healthy controls. **(A–C)** Boxplots of Chao1, Shannon, and Simpson diversity indices comparing alpha diversity between the two groups, with Shannon and Simpson indices showing statistically significant differences between groups (*P* < 0.001, ***); **(D)** Principal coordinate analysis (PCoA) based on unweighted UniFrac distance showing the separation trend of beta diversity between groups; **(E)** Principal coordinate analysis (PCoA) based on weighted UniFrac distance showing the separation trend of beta diversity between groups.

At the maximum sampling depth of 61,776 sequences, the mean Shannon index was significantly higher in the Control group (5.70 ± 0.06) compared to the ADHD group (5.28 ± 0.08), indicating greater microbial diversity in healthy controls. The flat trajectory of the curves beyond 7,000 sequences confirms that the sequencing depth was adequate for downstream alpha-diversity comparisons, and additional sequencing would not substantially increase the observed diversity. In the alpha-diversity analysis, we selected the Observed species, Chao1, Shannon, and Simpson indices to characterize species diversity and evenness within each sample (Figures 3A–C). Relative to the control group, the ADHD group exhibited a comparable Chao1 index (ADHD: 210 ± 37.0, median 210 [IQR 190–238]; Control: 205 ± 66.7, median 205 [IQR 187–275]) and Observed species (ADHD: 210 ± 37.0, median 210 [IQR 188–238]; Control: 205 ± 66.7, median 205 [IQR 185–272]), indicating preserved total species richness. However, the Shannon index was decreased in the ADHD group (5.15 ± 0.70, median 5.10 [IQR 4.85–5.90]) compared with controls (5.55 ± 0.22, median 5.55 [IQR 5.40–5.70]), as was the Simpson index (ADHD: 0.940 ± 0.022, median 0.942 [IQR 0.938–0.955]; Control: 0.950 ± 0.015, median 0.953 [IQR 0.948–0.955]). The significant reduction in Shannon and Simpson indices suggests decreased community evenness in the ADHD group. This pattern may result from multiple factors, including reduction of specific taxa, relative expansion of dominant groups, or combined shifts across multiple taxa. The unchanged Observed species index indicates that species richness was preserved, suggesting that the dysbiosis in ADHD is primarily characterized by community homogenization rather than simple overgrowth of dominant bacteria (Figure 2C).

To compare the gut microbial community structures between the ADHD and Control groups, we performed principal coordinate analysis (PCoA) based on two complementary distance metrics: Weighted UniFrac (Figure 3D) and Unweighted UniFrac (Figure 3E). The unweighted UniFrac PCoA (PC1 = 21.03%, PC2 = 11.27%) revealed substantial overlap between the ADHD (blue circles) and Control (red squares) groups, with no clear separation along either PC1 or PC2. The 95% confidence ellipses of the two groups intersected extensively, indicating that the two groups shared a largely similar repertoire of microbial species. In contrast, the weighted UniFrac PCoA (PC1 = 48.05%, PC2 = 18.71%) showed a more noticeable separation between the two groups, with ADHD samples distributing more broadly across the plot and Control samples concentrating in the central-left region. PERMANOVA confirmed significant group differences for both weighted UniFrac (F = 7.539, *P* = 0.001, R^2^ = 0.100) and unweighted UniFrac distances (F = 14.084, *P* < 0.001, R^2^ = 0.172). Notably, the effect size for unweighted UniFrac (R^2^ = 0.172) was larger than that for weighted UniFrac (R^2^ = 0.100), suggesting that both species presence/absence and abundance shifts contribute to the overall community differences, with rare taxa potentially playing a substantial role in distinguishing the two groups.

Our alpha-diversity analysis revealed that the ADHD group exhibited a comparable Chao1 index but decreased Shannon and Simpson indices relative to the Control group. This pattern – preserved species richness (Chao1) but reduced evenness and diversity (Shannon, Simpson) – is consistent with the beta-diversity findings. Collectively, these results suggest that ADHD is associated with altered gut microbial community structure characterized by preserved taxonomic richness but shifted abundance profiles. The reduced community evenness reflected by the decreased Shannon and Simpson indices could result from multiple factors, including the expansion of certain taxa, the reduction of others, or combined shifts across multiple taxa.

To intuitively visualize the composition and evolutionary distribution of high-abundance microbial taxa across all samples, we conducted a cladogram analysis based on the top 100 genera, focusing on the relative abundance shifts of these dominant genera at the phylum level. The results revealed that among phyla, Bacillota, shown in pink (denoting the highest abundance), exhibited the highest relative abundance, followed by *Bacteroidota* in sky blue (Figure 2D).

### 3.3 Gut microbiome profiling

**FIGURE 4:**
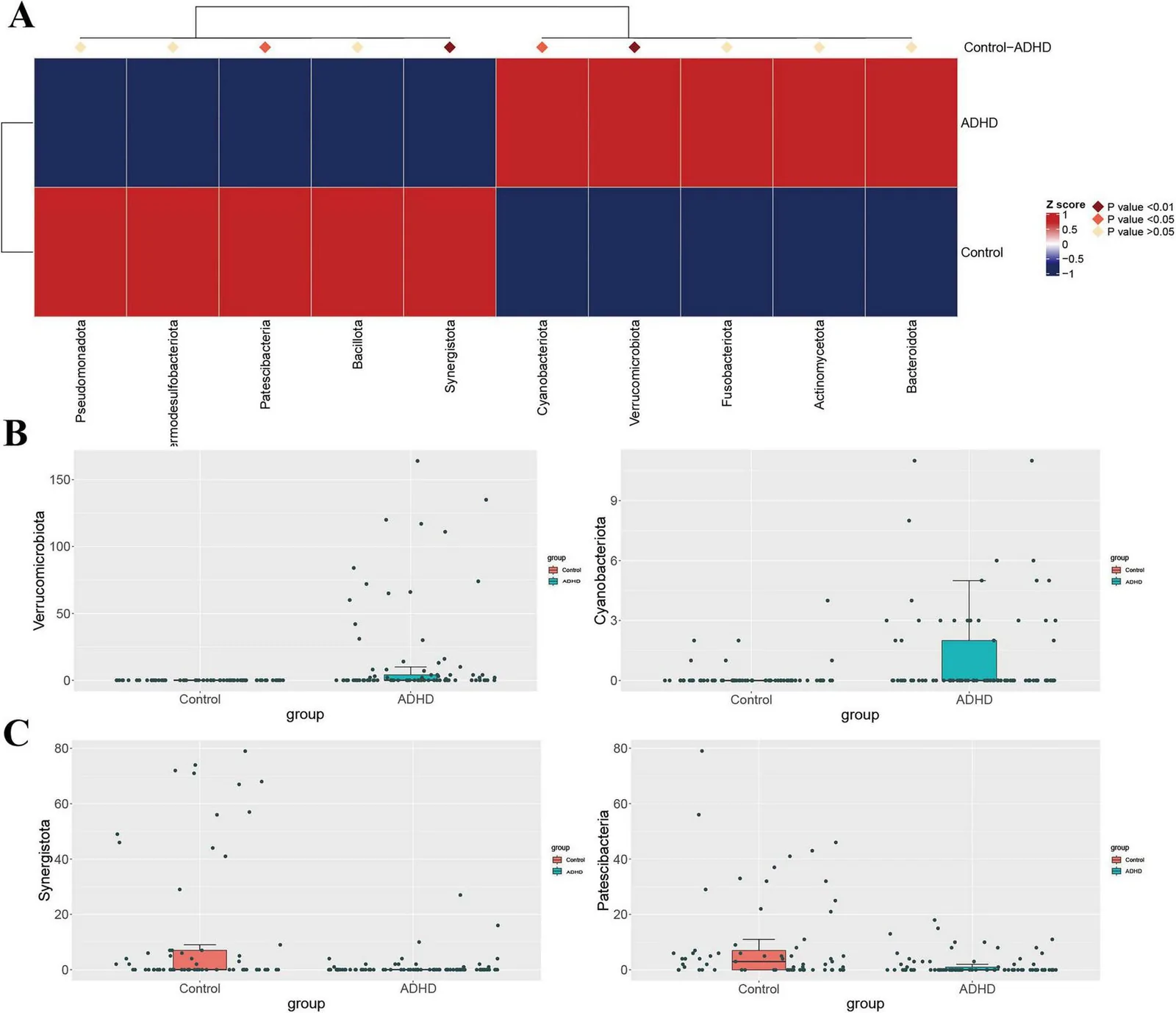
Differential abundance analysis at the phylum level between the ADHD group and the control group. **(A)** Heatmap of differentially abundant phyla based on the MetagenomeSeq method, with | logFC | >1 and *P* < 0.05 as the threshold for identifying significantly differential taxa. Statistical significance for each phylum is indicated above the heatmap: red diamonds denote *P* < 0.01, orange diamonds denote *P* < 0.05, and yellow circles denote *P* > 0.05. The color gradient (blue to red) represents Z score-standardized relative abundance (blue for low abundance, red for high abundance). Row clustering reveals the overall abundance pattern differences between the ADHD group and the Control group at the phylum level. **(B)** Scatter plots with boxplots showing individual sample relative abundances of phyla significantly enriched in the ADHD group: *Verrucomicrobia* (*P* = 1.51 × 10^−10^) and *Cyanobacteria* (*P* = 0.044). **(C)** Scatter plots with boxplots showing individual sample relative abundances of phyla significantly enriched in the Control group: *Synergistota* (*P* = 0.009) and *Patescibacteria* (*P* = 0.012).

To further evaluate phylum-level differences in microbial abundance between the ADHD and control groups, we performed differential abundance analysis using the MetagenomeSeq method (|logFC| > 1, *P* < 0.05). At the nominal level, the ADHD group showed higher abundances of *Cyanobacteriota* and *Verrucomicrobiota*, and lower abundances of *Synergistota* and *Patescibacteria*, relative to controls. Following Benjamini-Hochberg FDR correction, however, only *Verrucomicrobiota* remained statistically significant (adjusted *q* = 1.51 × 10^−10^), indicating robust enrichment in ADHD. *Cyanobacteriota* (*q* = 0.166), *Synergistota* (*q* = 0.060), and *Patescibacteria* (*q* = 0.060) did not withstand multiple-testing correction. These three phyla are therefore reported as nominally different at the uncorrected *P*-value level but not statistically significant after FDR adjustment (Figures 4A–C).

### 3.4 LEfSe analysis

**FIGURE 5:**
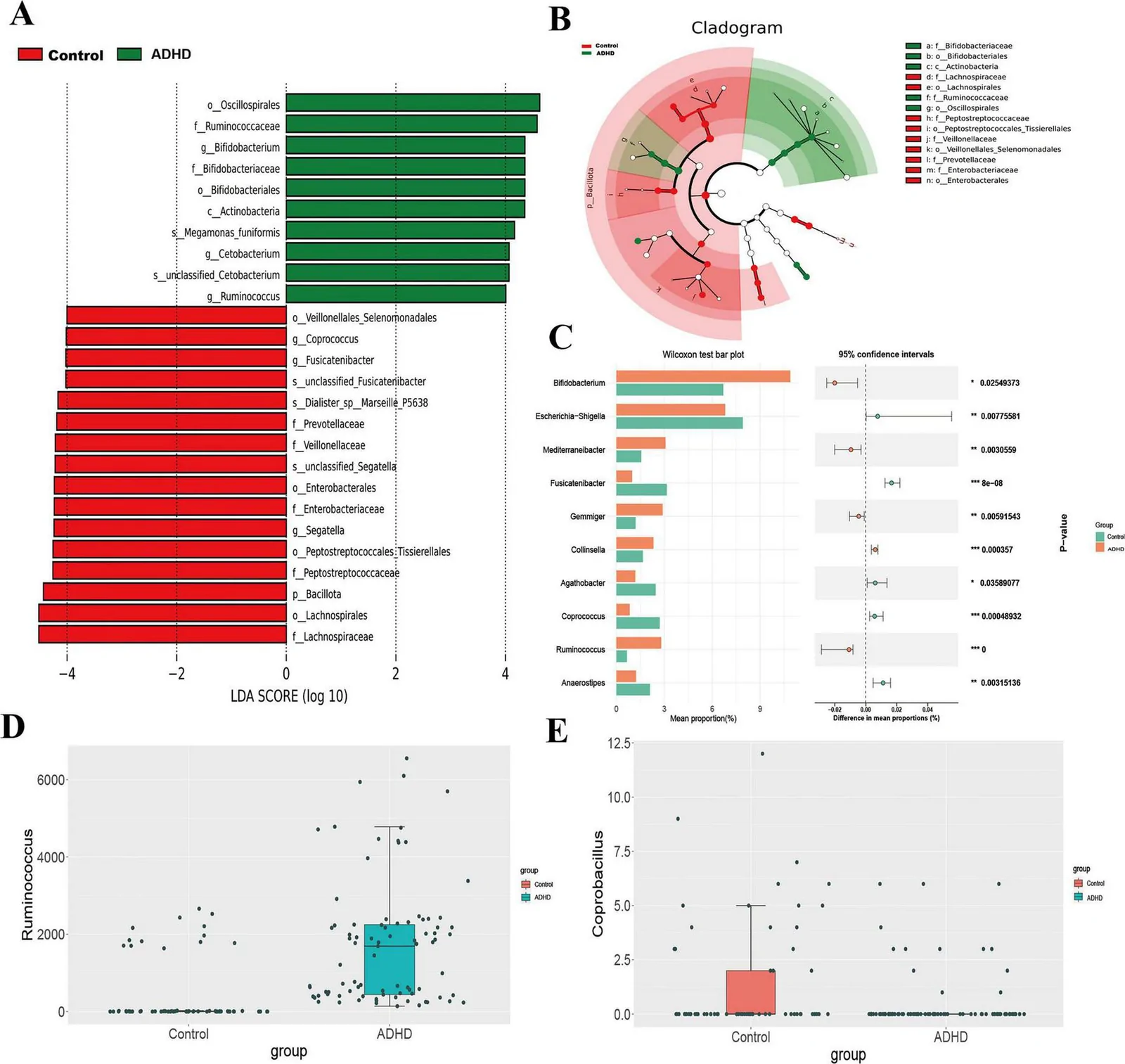
LEfSe analysis of differentially enriched taxa and validation of key differential genera between the ADHD group and the control group. **(A)** Linear discriminant analysis effect size (LEfSe) bar plot (LDA > 2, *P* < 0.05), with green bars indicating taxa significantly enriched in the ADHD group and red bars indicating taxa enriched in the control group; **(B)** Corresponding LEfSe cladogram showing the phylogenetic relationships of differentially enriched taxa from phylum to genus level, with green nodes and fan-shaped regions representing ADHD-enriched taxa and red representing control-enriched taxa; **(C)** Wilcoxon rank-sum test bar plot of mean proportions for differential genera with 95% confidence intervals, with *P* values and enriched groups annotated on the right (orange: ADHD group, green: control group); **(D, E)** Scatter plots with boxplots showing individual sample relative abundances of key differential genera (*Ruminococcus* and *Coprobacillus*), demonstrating that *Ruminococcus* was significantly elevated in the ADHD group (*P* < 0.05) **(D)**, while *Coprobacillus* was significantly enriched in the control group (*P* < 0.05) **(E)**.

To validate the aforementioned alterations at finer taxonomic resolutions, we performed LEfSe analysis to identify candidate biomarkers between the two groups. A total of 26 differentially abundant taxa were identified (Figure 5A), among which 10 taxa were significantly enriched in the ADHD group (LDA > 2.0, green) and 16 taxa in the Control group (LDA > 2.0, red). The characteristic microbiota of the ADHD group was predominantly concentrated within *Actinobacteria* and specific clades of *Bacillota*. At the phylum level, *Actinobacteria* emerged as a significant biomarker for the ADHD group. Progressing through the taxonomic hierarchy, the order *Bifidobacteriales*, family *Bifidobacteriaceae*, and genus *Bifidobacterium* all exhibited significant enrichment (LDA ≈ 4.0–4.5). Additionally, within *Bacillota*, the order *Oscillospirales*, family *Ruminococcaceae*, genus *Ruminococcus*, as well as *Megamonas funiformis* and *Cetobacterium* spp., were also significantly enriched in the ADHD group. In contrast, the characteristic microbiota of the Control group displayed a more extensive taxonomic distribution, encompassing multiple lineages within *Bacillota*, *Proteobacteria*, *Fusobacteriota*, and *Synergistetes*.

The cladogram further elucidated the phylogenetic clustering patterns of these differential taxa between the two groups (Figure 5B). Candidate biomarkers of the ADHD group were primarily clustered within the monophyletic branch of *Actinobacteria* and the *Ruminococcaceae* clade of *Bacillota*, exhibiting a relatively concentrated phylogenetic distribution. By comparison, biomarkers of the Control group were more dispersed across the phylogenetic tree, spanning multiple independent phylum-level lineages, suggesting a higher microbial diversity and more complex metabolic functional potential in the Control group.

To precisely validate and pinpoint differential taxa at the genus level, we employed three complementary statistical approaches for cross-validation: the Wilcoxon rank-sum test (Figure 5C), the *t*-test (Supplementary Figure 1), and metagenomeSeq differential abundance analysis (Figure 5D, E. Through multi-method consistency screening, *Ruminococcus* and *Coprobacillus* were ultimately identified as the most robust genus-level biomarkers distinguishing the two groups.

The Wilcoxon rank-sum test identified 10 differentially abundant genera (*P* < 0.05) (Figure 5C) In the ADHD group, *Bifidobacterium*, *Escherichia-Shigella*, *Mediterraneibacter*, *Gemmiger*, *Collinsella*, and *Ruminococcus* were significantly enriched. Among these, *Ruminococcus* exhibited the most pronounced intergroup difference, with its 95% confidence interval entirely deviating from zero and the largest effect size, indicating that this genus serves as a candidate biomarker for the ADHD group. Conversely, *Fusicatenibacter*, *Agathobacter*, *Coprococcus*, and *Anaerostipes* were significantly enriched in the Control group. To validate the robustness of the Wilcoxon test results, we further employed a parametric test (*t*-test) to compare genus-level abundances (Supplementary Figure 1). The *t*-test identified 36 differentially abundant genera (*P* < 0.01), substantially expanding the spectrum of discriminant microbiota. Highly consistent with the Wilcoxon results, *Ruminococcus* and *Mediterraneibacter* were significantly enriched in the ADHD group, whereas *Coprococcus*, *Anaerostipes*, and *Fusicatenibacter* were significantly enriched in the Control group.

To precisely lock in the candidate biomarkers suggested by both Wilcoxon and *t*-test results, we further employed metagenomeSeq for microbiome-specific differential abundance analysis to control for biases arising from sequencing depth and overdispersion. The intergroup distribution of *Ruminococcus* is shown in Figure 5D. In the Control group, the abundance of this genus was extremely low (median close to zero), whereas the ADHD group exhibited a marked enrichment with substantial inter-individual variability and multiple high-abundance outliers (up to > 6,000). The ADHD group displayed significantly greater interquartile range and overall range than the Control group, indicating highly heterogeneous colonization of this genus in ADHD patients. Conversely, *Coprobacillus* showed an entirely opposite distribution pattern (Figure 5E). Notably, after additional FDR correction, *Ruminococcus* remained highly significant (adjusted *q* < 1.0 × 10^−16^, effectively *q* ≈ 0), and *Coprobacillus* also passed the FDR threshold (adjusted *q* = 0.0016). Taken together, *Ruminococcus* and *Coprobacillus* exhibited the strongest enrichment signals in the ADHD and Control groups, respectively, and remained significantly different after both microbiome-specific correction by metagenomeSeq and FDR multiple-testing adjustment, thus being designated as tier-one candidate biomarkers.

## 4 Discussion

This study focuses on the gut microbiota characteristics of pre-pubertal females with ADHD and holds dual methodological significance. For a long time, the diagnostic rate of ADHD in females has been significantly lower than that in males; however, recent research suggests that this disparity is more likely attributable to insufficient clinical recognition of female phenotypes (predominantly inattentive presentation with fewer externalizing behaviors) rather than a true difference in prevalence (Mowlem et al., 2019; Young et al., 2020; Babinski, 2024; Solberg et al., 2025). This diagnostic bias has caused numerous girls to miss critical windows for early intervention, while the pre-pubertal stage represents both a key period of neurodevelopmental plasticity and an important node in the transition of gut microbiota toward an adult-like composition. In recent years, the mechanism by which gut microbiota participates in neuropsychiatric disorders through the “gut-brain axis” has received increasing attention. Microbial metabolites such as short-chain fatty acids, neurotransmitter precursors, and inflammatory mediators can influence central nervous system function via the vagus nerve, immune pathways, or the circulatory system (Dalile et al., 2019; Rutsch et al., 2020; Shaik et al., 2020; Silva et al., 2020; Ding et al., 2021; Liu et al., 2023; Caputi et al., 2021). In the field of ADHD, existing studies have suggested that dysbiosis may be associated with neuroinflammation (Han et al., 2021; Charitos et al., 2024; Costa and Cairrao, 2024), abnormal monoamine neurotransmitter metabolism (Garnock-Jones and Keating, 2009; Choi et al., 2024; Turiaco et al., 2024; Chu et al., 2025), and hypothalamic-pituitary-adrenal (HPA) axis dysfunction (Yan-Hui et al., 2009; Isaksson et al., 2012, 2013; Chang et al., 2020). However, most current microbiota research has focused on school-aged children or mixed-gender samples, failing to adequately control for the confounding effects of gonadal hormones on the microbiota-behavior association; systematic investigation of this specific population – pre-pubertal females – remains particularly scarce. From a developmental perspective, although pre-pubertal females are in a low-estrogen state, adrenarche has already been initiated, and subtle changes in androgen levels may influence microbial colonization through mechanisms not yet elucidated (Santos-Marcos et al., 2023; Cross et al., 2024; Guo et al., 2024; Lei et al., 2024). Meanwhile, this stage has not yet been strongly subjected to the typical cyclical fluctuations of gonadal hormones, so microbiota differences observed at this time more accurately reflect “original” sex-related characteristics rather than adaptive changes secondary to dramatic hormonal shifts. Therefore, by selecting this unique window that is “hormonally quiescent yet developmentally active,” this study aims to exclude confounding effects of pubertal hormonal fluctuations, characterize potential microbial signatures associated with female ADHD, and inform future sex-specific research into microbiota-behavior relationships.

Our diversity analyses revealed a distinctive alteration in the gut microbial community structure among pre-pubertal females with ADHD, characterized by preserved species richness but reduced community evenness – a pattern indicative of microbial community destabilization. This reduced evenness, coupled with increased within-group heterogeneity in the ADHD group, suggests that the disorder-associated microbial shift may operate through two interrelated mechanisms: the depletion or loss of rare taxa, and an unstable abundance distribution among dominant members. The fact that unweighted beta-diversity metrics, which are sensitive to rare taxa, showed a more pronounced group separation than weighted metrics further supports the view that rare taxa loss is a primary driver of community divergence in ADHD. From a functional perspective, such alterations in community evenness and dispersion may reflect impaired ecological resilience of the gut ecosystem, potentially compromising its capacity to resist perturbations or maintain metabolic homeostasis. These compositional changes, particularly the reduction in community evenness, may have functional consequences for the host, given that reduced evenness has been associated with altered metabolic output and inflammatory status in previous studies. Taken together, our findings suggest that ADHD in pre-pubertal females is associated with a structurally reorganized but not simply species-depleted gut microbiota, with the imbalance between dominant and rare members potentially serving as an ecological basis for further functional alterations.

The phylum-level analysis revealed that only *Verruco microbiota* remained significantly enriched in the ADHD group after multiple-testing correction, with *Akkermansia muciniphila* as its predominant representative. Given the well-characterized role of *A. muciniphila* in mucin degradation and intestinal barrier maintenance (Paone and Cani, 2020; de Vos et al., 2022), its elevated abundance in our ADHD cohort is intriguing. However, without concurrent metabolomic or mucin turnover data, we cannot ascertain whether this enrichment reflects a compensatory barrier-reinforcing response or an alternative ecological adaptation; this warrants targeted investigation in future studies. Notably, several other phyla – including *Cyanobacteriota*, *Synergistota*, and *Patescibacteria* – showed trends toward differential abundance prior to correction but did not survive FDR adjustment, suggesting that their apparent signals may be driven by individual variation or insufficient statistical power rather than true biological effects.

At the genus level, cross-validation across LEfSe, metagenomeSeq, Wilcoxon rank-sum, and *t*-test consistently identified *Ruminococcus* and *Coprobacillus* as robust differential taxa, with both remaining significant after FDR correction. Notably, we did not assess associations between the abundance of these taxa and ADHD symptom severity scores; therefore, the following interpretations address group-level differences only and should not be construed as evidence of dose-response relationships with clinical phenotype.

*Ruminococcus* showed significant enrichment in the ADHD group and remained statistically significant after metagenomeSeq correction. As a core member of the *Ruminococcaceae* family within the phylum Firmicutes, this genus is a major cellulose-degrading bacterium and butyrate producer in the gut. Butyrate, as the primary energy source for colonic epithelial cells, plays a key role in maintaining intestinal barrier integrity, regulating immune responses, and suppressing neuroinflammation (Martínez de Victoria Carazo et al., 2023; Schaus et al., 2024). However, we did not measure butyrate levels or intestinal barrier function, and the following interpretations are therefore speculative. The elevation of *Ruminococcus* in this study may, in theory, reflect the following hypothesized mechanisms. First, ADHD patients might have altered dietary fiber intake patterns or prolonged intestinal transit time, leading to compensatory proliferation of this genus (Wu et al., 2011; Hills et al., 2019; Paul et al., 2025). Second, overproliferation of *Ruminococcus* could potentially competitively inhibit the colonization of other butyrate-producing bacteria. If *Ruminococcus* has a lower butyrate yield per cell than the displaced species, this competition could theoretically paradoxically result in decreased net butyrate production and increased intestinal barrier permeability, forming a “high-abundance, low-function” paradox (Huang et al., 2021; Shah et al., 2023). Third, metabolites of this genus can modulate vagal afferent signals by activating free fatty acid receptors on intestinal epithelial cells, thereby influencing HPA axis stress responses (Clark and Mach, 2016; Hamamah et al., 2022; Juárez-Fernández et al., 2023; Wang et al., 2024; Zhang et al., 2025). If these pathways are operative in humans, perturbation of *Ruminococcus* might have long-term programming effects on stress sensitivity during this developmental window; however, this remains entirely conjectural without functional validation.

In contrast to *Ruminococcus*, *Coprobacillus* was significantly enriched in the control group and was nearly completely absent in the ADHD group. This genus belongs to the *Erysipelotrichaceae* family and is closely associated with bile acid metabolism, lipid metabolism, and host energy homeostasis. The following pathways are proposed as hypotheses only. The absence of *Coprobacillus* has been hypothesized to relate to ADHD pathology through the following pathways. First, this genus is involved in the biotransformation of primary bile acids to secondary bile acids. Its absence may lead to alterations in the secondary bile acid profile, thereby regulating systemic metabolism through the G protein-coupled bile acid receptor (TGR5), potentially affecting neurodevelopment of the prefrontal-striatal circuit (Yuan et al., 2020; Cao et al., 2024; Feng et al., 2024; Misiak et al., 2024; Berendse et al., 2026). Second, the lipid metabolic function of *Coprobacillus* may indirectly affect substrates for sex hormone synthesis (such as cholesterol metabolism). The subtle changes in androgens initiated by adrenarche during the pre-pubertal stage may participate in the sex-specific pathological expression of female ADHD through the lipid-hormone metabolic axis (Yuan et al., 2020, 2022; Park et al., 2022; Zhang et al., 2022; Yang et al., 2024). Finally, the absence of this genus may reflect systematic changes in the gut microenvironment of ADHD patients, leading to a cascading loss of colonization resistance and exacerbating the vicious cycle of dysbiosis (Yuan et al., 2020). These interpretations await validation through metabolomic and functional studies.

This study has several limitations. First, the cross-sectional design precludes causal inference; we cannot determine whether microbial differences are a cause or consequence of ADHD. Second, the sample size is small, precluding stratified analyses by subtype, medication, or diet. Third, dietary intake, physical activity, and socioeconomic status were unmeasured and unadjusted, limiting attribution of observed differences to ADHD specifically. Fourth, 16S rRNA sequencing cannot resolve *Ruminococcus* to the species level, and functional interpretations remain speculative. Fifth, we measured neither butyrate, bile acids, HPA axis markers, nor androgens; mechanistic interpretations for *Ruminococcus* and *Coprobacillus* are therefore hypothetical and may reflect unmeasured confounders rather than ADHD-specific biology. The “sex-specific” framing is also unsupported, as no cross-sex comparison was performed. Sixth, symptom severity scores were not correlated with microbial taxa, precluding assessment of dose-response relationships. Finally, single time-point sampling may miss dynamic changes. In addition, stool consistency was not assessed at sample collection, precluding adjustment for intestinal transit time as a potential confounder of microbiota composition.

In our ongoing and future work, we will pursue the following priorities: (i) longitudinal designs to establish temporal directionality; (ii) measurement and statistical adjustment of dietary, physical activity, and socioeconomic confounders; (iii) metagenomic and metabolomic profiling to validate functional mechanisms; (iv) correlation analyses between taxa and symptom dimensions; and (v) cross-sex comparisons before any sex-specific claims. Intervention studies are premature until these foundational questions are addressed.

## 5 Conclusion

We found that pre-pubertal females with ADHD have higher *Ruminococcus* and lower *Coprobacillus*, with reduced gut bacterial evenness but unchanged richness. This points to a reshuffling within specific microbial lineages, not wholesale species loss, and may influence ADHD via the gut-brain axis. However, these cross-sectional associations are confounded by unmeasured dietary, lifestyle, and socioeconomic factors, and we did not assess symptom severity correlations or functional mechanisms. Ongoing and future work will employ longitudinal designs, multi-omic profiling, and cross-sex comparisons to determine whether these microbial signatures represent causal contributors, consequences of ADHD-related behaviors, or correlates of unmeasured confounders.

## Data Availability

The data presented in this study can be found in the GSA database (https://ngdc.cncb.ac.cn/gsa/) under accession number PRJCA069535.
