# Supplementary material for: Gut microbiota profiles and potential biomarkers in pre-pubertal females with ADHD
Source: Front Microbiol. 2026 Aug 20;17:1894383. doi: 10.3389/fmicb.2026.1894383 (PMC13540380; doi:10.3389/fmicb.2026.1894383)
Supplement: Supplementary file 1 [file Supplementary_file_1.docx]

**Fig S1. T-test-based differential abundance analysis at the genus level between the ADHD and control groups.** The left panel shows mean relative abundance bar plots for differentially abundant genera (orange: control group; blue: ADHD group); the right panel displays 95% confidence intervals of mean differences between groups with corresponding *P* values. *Ruminococcus* and *Mediterraneibacter* were significantly enriched in the ADHD group, whereas *Coprococcus*, *Anaerostipes*, and *Fusicatenibacter* were significantly enriched in the control group. A total of 36 differentially abundant genera were identified (*P* < 0.01).
