# Supplementary material for: Gut microbiota profiles and potential biomarkers in pre-pubertal females with ADHD
Source: Front Microbiol. 2026 Aug 20;17:1894383. doi: 10.3389/fmicb.2026.1894383 (PMC13540380; doi:10.3389/fmicb.2026.1894383)
Supplement: Supplementary file 2 [file Supplementary_file_2.docx]

**Supplementary Table S1: Alpha-Diversity Metrics (Mean ± SD and Median [IQR]) by Diagnostic Group**

| Index | Group | Mean ± SD | Median (IQR) |
| --- | --- | --- | --- |
| Shannon | ADHD | 5.15 ± 0.70 | 5.10 (4.85–5.90) |
|  | Control | 5.55 ± 0.22 | 5.55 (5.40–5.70) |
| Simpson | ADHD | 0.940 ± 0.022 | 0.942 (0.938–0.955) |
|  | Control | 0.950 ± 0.015 | 0.953 (0.948–0.955) |
| Chao1 | ADHD | 210 ± 37.0 | 210 (190–238) |
|  | Control | 205 ± 66.7 | 205 (187–275) |
| Observed Features | ADHD | 210 ± 37.0 | 210 (188–238) |
|  | Control | 205 ± 66.7 | 205 (185–272) |

**Supplementary Statistical Validation of Beta-Diversity Analysis**

To rigorously evaluate group differences in microbial community structure, we performed PERMANOVA with 9,999 permutations on both Unweighted and Weighted UniFrac distance matrices. Both analyses confirmed statistically significant differences between the Control (n = 27) and ADHD (n = 43) groups (Table S1): Unweighted UniFrac showed the strongest effect (F = 14.084, *P* < 0.001, R² = 0.172), while Weighted UniFrac also reached significance (F = 7.539, *P* = 0.001, R² = 0.100). The higher R² value for Unweighted UniFrac suggests that differences in the presence or absence of rare taxa contribute substantially to the observed group separation, despite the visual overlap in the PCoA plot.

To ensure that the PERMANOVA results were not confounded by differences in within-group dispersion, we performed PERMDISP tests (Table S3). For Unweighted UniFrac, dispersion was homogeneous between groups (F = 0.809, *P* = 0.382; Control dispersion = 0.392, ADHD dispersion = 0.377), validating that the significant PERMANOVA result reflects genuine centroid differences rather than unequal variability. In contrast, Weighted UniFrac exhibited significant heterogeneity of dispersion (F = 110.579, *P* < 0.001; Control dispersion = 0.189, ADHD dispersion = 0.288), indicating that the ADHD group harbors substantially greater inter-individual microbiome variability than Controls. Consequently, the significant PERMANOVA result for Weighted UniFrac should be interpreted cautiously: the observed group separation may be driven by both centroid location differences and higher within-group dispersion in ADHD. This elevated heterogeneity in the ADHD group is consistent with the decreased Shannon and Simpson indices observed in our alpha-diversity analysis, which together suggest that ADHD is characterized by disrupted microbial community evenness and increased inter-subject variability in taxon abundance profiles.

PERMANOVA Results (Table S2)

| Distance Metric | F-statistic | *p*-value | R² | Interpretation |
| --- | --- | --- | --- | --- |
| Unweighted UniFrac | 14.084 | 0.0001 | 0.1716 | Highly significant group separation |
| Weighted UniFrac | 7.539 | 0.0010 | 0.0998 | Highly significant group separation |

Homogeneity of Dispersion Tests (PERMDISP) (Table S3)

| Distance Metric | F-statistic | *p*-value | Control Dispersion | ADHD Dispersion | Assessment |
| --- | --- | --- | --- | --- | --- |
| Unweighted UniFrac | 0.809 | 0.382 | 0.392 | 0.377 | Homogeneous |
| Weighted UniFrac | 110.579 | 0.0001 | 0.189 | 0.288 | Heterogeneous |
